# N-Glycosylation and N-Glycan Processing in HBV Biology and Pathogenesis

**DOI:** 10.3390/cells9061404

**Published:** 2020-06-04

**Authors:** Mihaela-Olivia Dobrica, Catalin Lazar, Norica Branza-Nichita

**Affiliations:** Institute of Biochemistry of the Romanian Academy, Splaiul Independentei 296, Sector 6, 060031 Bucharest, Romania; olivia@biochim.ro (M.-O.D.); clazar@biochim.ro (C.L.)

**Keywords:** HBV, glycosylation, folding, vaccine, immune response, HCC

## Abstract

Hepatitis B Virus (HBV) glycobiology has been an area of intensive research in the last decades and continues to be an attractive topic due to the multiple roles that N-glycosylation in particular plays in the virus life-cycle and its interaction with the host that are still being discovered. The three HBV envelope glycoproteins, small (S), medium (M) and large (L) share a very peculiar N-glycosylation pattern, which distinctly regulates their folding, degradation, assembly, intracellular trafficking and antigenic properties. In addition, recent findings indicate important roles of N-linked oligosaccharides in viral pathogenesis and evasion of the immune system surveillance. This review focuses on N-glycosylation’s contribution to HBV infection and disease, with implications for development of improved vaccines and antiviral therapies.

## 1. Introduction

Attachment of oligosaccharides to Asn residues within consensus sequences (Asn-X-Ser/Thr) of nascent polypeptides, also known as N-linked glycosylation, is both a highly conserved and the most abundant protein modification occurring in the endoplasmic reticulum (ER) of eukaryotic cells [1]. Therefore, it is not surprising that this process has crucial consequences on the fate of resulting glycoproteins by regulating their folding into the native conformation, trafficking and stability.

Most enveloped viruses take advantage of this host-specific pathway to glycosylate own surface proteins, with important consequences at different stages of their lifecycles.

**Figure 1:**
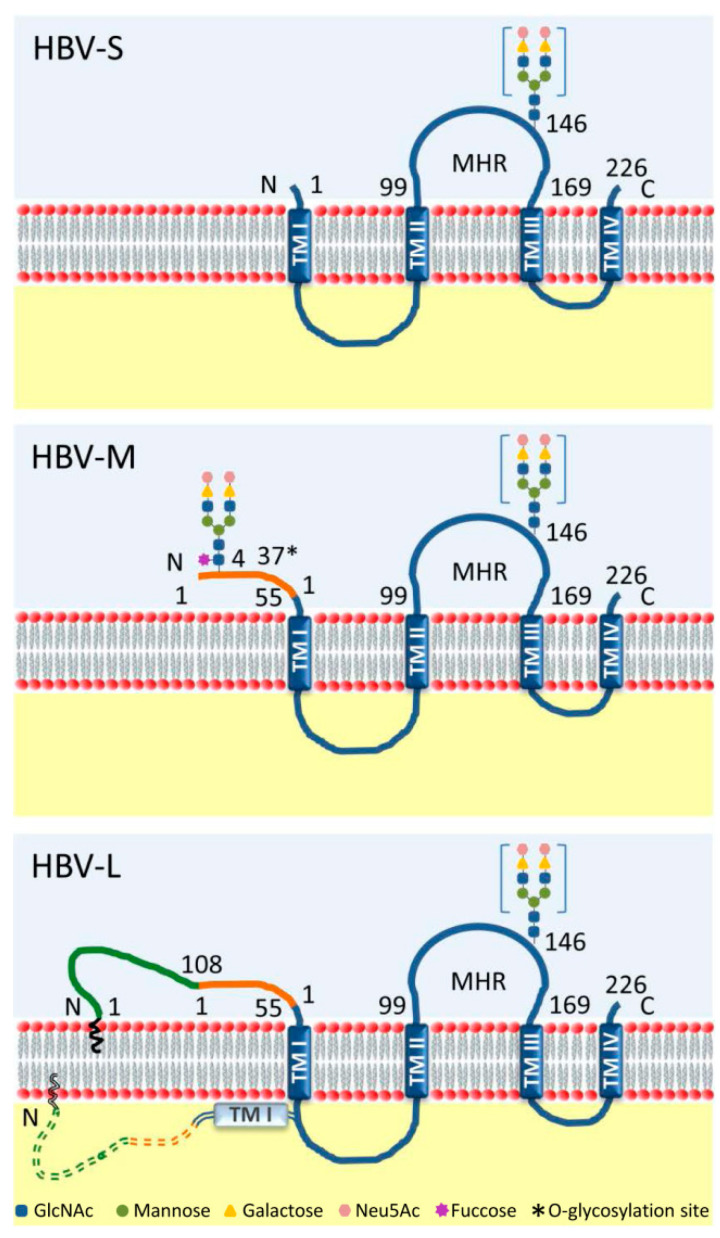
Schematic representation of the Hepatitis B Virus (HBV) envelope glycoproteins. S, M and L proteins contain four transmembrane domains (TM I-IV) and share a common S domain (blue). HBV-M is extended with the preS2 domain (orange) at the N-terminus, while HBV-L has an additional pre-S1 domain (green). HBV-L is characterized by a dual topology of the pre-S region, facing either the ER lumen (solid line) or the cytosol (dashed line). The two functional N-glycosylation sites are indicated: N4 in the preS2 region, occupied only in HBV-M; and N146 in the major hydrophilic region (MHR) of the S domain, occupied in half (in square brackets) of all three proteins [6]. The complex structure of the N-glycans is represented [10,11]. The O-glycosylation site identified in the preS2 domain of HBV-M is also shown (*) [10].

Hepatitis B Virus (HBV) is a human pathogen and a member of the *Hepadnaviridae* family of DNA viruses with liver tropism. HBV infection causes the death of nearly 1 million people every year, while a cure is still missing [2]. HBV-infected hepatocytes produce 42 nm infectious virions, consisting in a nucleocapsid protected by a lipid membrane harboring the small (S), medium (M) and large (L) surface (envelope) glycoproteins. These transmembrane proteins are translated from the same open reading frame (ORF) and have a common carboxy-terminal end, corresponding to the S sequence. M has an additional pre-S2 domain, while L extends M by the pre-S1 polypeptide (Figure 1). A remarkable property of these proteins is the ability to self-associate at the ER membrane into nucleocapsid-free subviral particles (SVPs), collectively denoted as HBV surface antigens (HBsAg). Depending on the S-to-L ratio during morphogenesis, SVPs are produced in either spherical or filamentous shapes. Spheres are about 25 nm in diameter and contain comparable amounts of S and M and only traces of L. Co-incorporation of larger amounts of L results in assembly of 22-nm diameter filaments of different lengths [3,4]. Production of HBsAg by infected cells largely exceeds that of virions, possibly as an adaptive mechanism to neutralize the host immune response against infectious HBV particles [5].

Although not heavily glycosylated, the HBV envelope proteins exploit the host N-glycosylation pathway in a very peculiar manner. All three proteins share a potential N-glycosylation site at Asn-146 (N146) of the S domain; however, this is functional in about half of all envelope proteins, resulting in similar amounts of glycosylated and non-glycosylated S, M and L isoforms (Figure 1). A second potential N-glycosylation site at Asn-4 (N4) of the pre-S2 domain is always occupied in M, but not L, most probably due to the latter adopting a dual topology and exposing this domain both in the cytoplasm and the ER lumen [6,7] (Figure 1). These sites are conserved among all HBV genotypes, indicating instrumental roles in biosynthesis and function of the envelope proteins [8]. In addition to N-glycosylation, pre-S2 domains of M proteins from HBV genotypes C and D may also be O-glycosylated [9,10].

For more than two decades since the first sequencing of the HBV N-linked glycans, viral glycosylation has been the subject of intensive investigation. While many features of the HBV life-cycle have been undoubtedly associated with this process, other important roles of N-glycosylation in viral pathogenesis and evasion of the immune response are currently emerging.

This review aims to reveal the intricate mechanisms by which carbohydrates attached to the HBV envelope proteins regulate HBV infection and contribute to disease.

## 2. Trimming of HBV N-glycans by the ER α-glucosidases I and II: Asset or Vulnerability?

**Figure 2:**
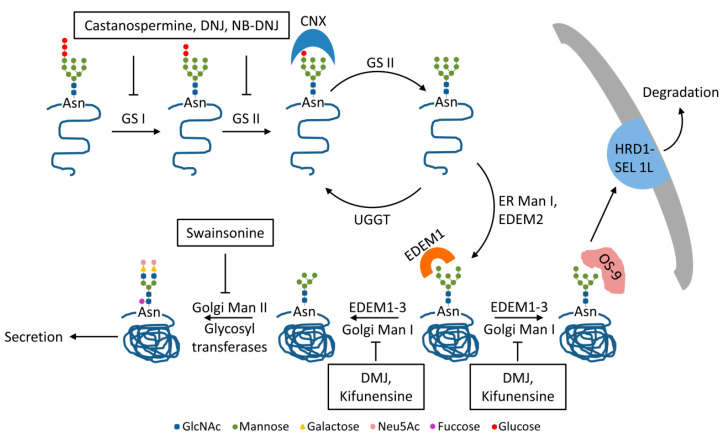
N-glycan trimming of the HBV envelope glycoproteins. Following the attachment of the (GlcNAc)_2_Man_9_Glc_3_, precursor to nascent viral polypeptides, the ER α-glucosidase I (GS I) removes the terminal Glc, followed by ER α-glucosidase II (GS II) trimming the second Glc unit. The mono-glucosylated glycoproteins enter the protein folding cycle assisted by ER-resident chaperones [13,14]. Interaction with calnexin (CNX) was demonstrated for M and L proteins [16,17]. Subsequent to trimming of the last Glc unit by GS II, improperly folded proteins are re-glucosylated by UDP-glucose:glycoprotein glucosyltransferase (UGGT) and reenter the cycle. Correctly folded glycoproteins are subjected to further mannose (Man) trimming by ER mannosidase I (ER Man I), followed by Golgi mannosidases I and II. Finally, the N-glycan is processed by a series of glycosyl transferases, leading to complex structures on secreted glycoproteins [13,14]. The ER degradation-enhancing, mannosidase-like proteins (EDEMs )are also responsible for Man trimming, leading to Man_5-7_ intermediates. S and L proteins are substrates for the OS-9 lectin that delivers them to HRD1-SEL 1L complex for degradation. In contrast, EDEM trimming accelerates M protein trafficking through the secretory pathway [18,19]. Specific inhibitors for the N-glycan trimming steps are indicated [14,18].

Once transferred from the lipid donor to Asn residues within consensus sequences of the viral proteins by the oligosaccharyl transferase, the (GlcNAc)_2_Man_9_Glc_3_ precursor is subjected to a series of modifications by ER- and Golgi-resident enzymes [12]. The N-glycan trimming is a key process in the quality control of glycoprotein folding. It is initiated by the ER α-glucosidase I, which cleaves the terminal α1-2-linked glucose (Glc) unit from the (GlcNAc)_2_Man_9_Glc_3_ oligosaccharide. The next two α1-3-linked Glc moieties are further removed by the ER α-glucosidase II, resulting in the (GlcNAc)_2_Man_9_ glycan structure [13,14] (Figure 2). Trimming of the terminal Glc residues from the initial N-linked oligosaccharide provides the substrates for calnexin/calreticulin-assisted folding. These two ER-resident lectins specifically interact with mono-glucosylated poly-mannose glycans attached to protein folding intermediates of both cellular and viral origin, preventing potential aggregation and premature degradation. Removal of the last Glc unit releases glycoproteins from the calnexin/calreticulin cycle regardless of their conformation. While correctly folded proteins become substrates to subsequent N-glycan trimming along the secretory pathway, polypeptides with difficulties to attain the native structure are recognized by UDP-glucose:glycoprotein glucosyltransferase (UGGT) and re-glucosylated to enable further folding within the lectins cycle [13,15] (Figure 2). It is interesting to note that for most glycoproteins, transient binding to calnexin or calreticulin, while clearly beneficial for their folding efficiency, is not critical for the acquirement of the native conformation.

A first, albeit indirect, evidence of a role of the calnexin/calreticulin cycle in the folding of the HBV glycoproteins resulted from studies using specific ER α-glucosidases I and/or II inhibitors, such as the iminosugars deoxynojirimycin (DNJ) or the alkylated derivative, n-butyl-deoxynojirimycin (NB-DNJ). These inhibitors strongly prevented HBV, but not SVP secretion [20,21]. Similar effects were found in the presence of tunicamycin, which inhibits N-linked glycosylation by preventing addition of the precursor oligosaccharide to nascent polypeptides [21,22]. Since the S protein is the major constituent of both types of HBV particles, while L and M are enriched in virions, the data indicated that the envelope proteins have distinct requirements for N-glycosylation and trimming with regard to their folding and assembly [21]. Further analysis demonstrated an aberrant trafficking of the HBV glycoproteins in the presence of NB-DNJ. While the drug promoted intracellular retention of M within the ER–Golgi intermediate compartments, S and L were not affected by the treatment [23]. Moreover, secreted SVPs containing only S and L proteins displayed fully processed glycans, suggesting they were able to bypass the inhibition of α-glucosidases I and II activities [11]. It was later shown that M, and to a lesser extent L, was subjected to proteasome-mediated degradation in the presence of NB-DNJ, even though ubiquitination of these proteins could not be demonstrated [24,25]. Since the proteasome inhibition failed to completely reverse the observed degradation, the process may involve other cellular disposal sites, such as the lysosomes, as suggested in previous studies [23].

Further confirmation of the unusual dependence of the M protein on N-glycosylation was provided by studies using HBV variants with mutated N-glycosylation consensus sites. Removal of the preS2, but not of the common N-linked glycan within the S domain, significantly inhibited virion secretion, indicating a previously unrecognized role of the M protein in HBV egress. Thus, although M itself is dispensable for HBV assembly and secretion, the preS2 glycan promotes M as a major regulator of virion trafficking, when the protein is present on the viral envelope [11]. The molecular mechanism underlying this unusual behavior was established following investigation of the HBV envelope proteins folding and interaction with the calnexin/calreticulin cycle [16]. A key discovery of this work is that the N4 glycan is responsible for M binding to calnexin, as demonstrated by co-immunoprecipitation assays. In addition, the study revealed that secretion of M-SVPs is strictly dependent on the occupancy of the N4 glycosylation site, while the common glycan is dispensable. Together, these findings strongly suggest a functional role for calnexin in supporting M protein folding, mediated by the preS2 N4 glycan. In contrast, the S protein does not associate with calnexin, and the lack of the N146 glycan does not affect secretion of S-SVPs [16]. Therefore, the drastic effect of α-glucosidases inhibitors on HBV secretion that had been observed previously could be accounted for by a lack of interaction of M with calnexin, in the presence of the drugs, resulting in M misfolding, degradation and possibly viral envelope instability.

Interestingly, the L protein was also found to bind calnexin, despite the protein lacking the N4-linked glycan in the preS2 domain [10,26]; however, in this case, the interaction appears to retain the L polypeptide in a pre-Golgi compartment [17]. This observation is supported by the results obtained in DNJ-treated, HBV-replicating cells, indicating an impaired viral secretion, accompanied by a reduced intracellular level of both L and M proteins [24]. This intriguing cellular retention of L by calnexin may be a mechanism adopted by HBV to prevent secretion of L-SVPs and avoid potential competition with virus particles for receptor binding and infection of target cells [17].

An important question remaining to be addressed regarded the role of N-linked glycans in HBV infectivity. These investigations became feasible with the development of reliable cell culture systems able to support HBV infection, a notoriously difficult process in vitro [27]. One approach employed treatment of HBV-replicating, HepG2.2.15 cells with NB-DNJ, to inhibit the ER α-glucosidases I and II, arrest the N-glycan trimming and thus prevent interaction of the M protein with calnexin [28]. As expected, NB-DNJ significantly inhibited HBV secretion in a dose-dependent manner. At higher drug concentration, the M protein became undetectable in samples of sucrose-concentrated virus, which correlated with a loss of HBV infectivity by 80%, as demonstrated in HepaRG cells [28]. It was proposed that misfolding and degradation of M during treatment led to a significant change of L:M ratio on the virus envelope, affecting the viral stability. These results appeared surprising, as previous data have shown that N-glycans of HBV envelope proteins were not required for the HBV satellite, Hepatitis Delta Virus (HDV), entry into primary hepatocytes [29]. However, the mutational studies performed in the work mentioned above did not examine the pre-S2 N-glycan, as the M protein was considered dispensable for HBV infection and hence omitted from the analysis [29].

Since the discovery of the role played by the ER α-glucosidases I and II in the HBV life-cycle, these enzymes have become appealing targets for the development of antiviral therapies, and substantial effort has been made to improve the antiviral efficiency of the lead inhibitors. More than 200 DNJ derivatives with diverse side-chains have been chemically synthesized and tested against a variety of other enveloped viruses encoding N-linked glycoproteins [30]. Of these, N-nonyl-DNJ (NN-DNJ), was able to reduce viremia in woodchucks infected with an HBV surrogate model, woodchuck hepatitis virus (WHV) [31]. However, the glucosidase inhibitors currently available still need improvement, as administration of high doses is required to achieve a robust antiviral response. In addition, these drugs often have side effects. Future studies should take advantage of the recently disclosed ER-α-glucosidases structures and rationally design novel chemical modification of the current scaffolds to enhance the inhibitory activity as well as cellular delivery and push these drugs into clinical trials [32,33].

## 3. Mannose Trimming of HBV N-glycans: Crossroads between Protein Trafficking and Degradation

After being released from the calnexin/calreticulin cycle, correctly folded proteins are subjected to further N-glycan processing [13]. The ER α-mannosidase I catalyzes the trimming of the terminal α1-2-linked mannose (Man) residue from the middle chain. The resulting (GlcNAc)_2_Man_8_-bound polypeptide is transported to cis-Golgi, where the Golgi α-mannosidase I removes the remaining three α1-2-linked Man units, with formation of (GlcNAc)_2_Man_5-7_ intermediates [13,14]. This trimming provides the substrates for subsequent processing by Golgi α-mannosidase II and various glycosyl-transferases in medial and trans-Golgi compartments, resulting in the complex N-glycan structures of mature viral particles and SVPs leaving the cell via multivesicular bodies (MVBs) and the constitutive secretory pathway, respectively [26]. N-glycan sequencing of patient-derived HBV particles (genotypes A, C and D) has revealed the presence of partially sialylated, biantennary (Galβ4GlcNAcβ2Manα3[Galβ4GlcNAcβ2Manα6] Manβ4-GlcNAcβ4GlcNAc) complex-type glycans [10]. Addition of a fucose unit on the N4 glycan was observed in particles produced in tissue culture [11] (Figure 2).

Some glycoproteins also undergo early mannose trimming by ER degradation-enhancing, mannosidase-like proteins, EDEM 1, 2 and 3. EDEMs belong to a family of proteins with mannosidase activity that are strongly up-regulated during the unfolded protein response (UPR), a network of pathways activated by the cell to relieve the ER stress [34]. Removal of up to four Man residues from the N-linked oligosaccharides targets the glycoproteins to the ER-associated degradation (ERAD) to reduce the protein burden and alleviate the stress condition. EDEM proteins have partially overlapping substrate specificity. EDEM2 acts on (GlcNAc)_2_Man_9_ to form (GlcNAc)_2_Man_8_ oligosaccharides. These are further trimmed by EDEM1 and/or EDEM3, leading to (GlcNAc)_2_Man_5-7_ intermediates that are taken over by the OS-9 lectin and delivered to proteasomal degradation [35,36] (Figure 2).

We and others have shown that HBV replication triggers ER stress, UPR activation and significant up-regulation of all members of the EDEM family [37,38]. Massive reduction of intracellular levels of S, M and L envelope proteins was observed in cells transiently over-expressing EDEM 1, 2 or 3 [18,38]. The data indicated that S and L were degraded in the presence of EDEMs, accompanied by a decrease of secreted SVPs. Endogenous EDEMs silencing not only reversed this phenotype but greatly improved secretion of S-SVPs, suggesting that a large amount of wild-type, rather than misfolded HBV envelope proteins are subjected to degradation during the course of infection. The process also involves suppressor of lin-12-like 1 SEL 1L, another ERAD component, as very recently proposed [19] (Figure 2).

In striking contrast, M was rapidly trafficked through the secretory pathway, and secretion of M-SVPS significantly increased from cells overexpressing EDEMs [18]. The intriguing behavior of the M protein strictly depended on the N-glycan processing by EDEMs, as confirmed by using the mannosidases inhibitors deoxymannojirimycin (DMJ) and kifunensine. Moreover, the use of glycosylation mutants revealed that the preS2 glycan is responsible for the specific effect of EDEMs on M secretion [18]. This property appears particularly relevant in the context of ER stress, as secretion of HBV particles lacking the M protein is greatly reduced in cells treated with pharmacological UPR inducers [18]. This observation shed more light on the disputed role of the M envelope protein in the HBV life-cycle, providing an explanation for the enigmatic maintenance of this protein in the viral genome, despite the clearly demonstrated vulnerability as antiviral target.

**Figure 3:**
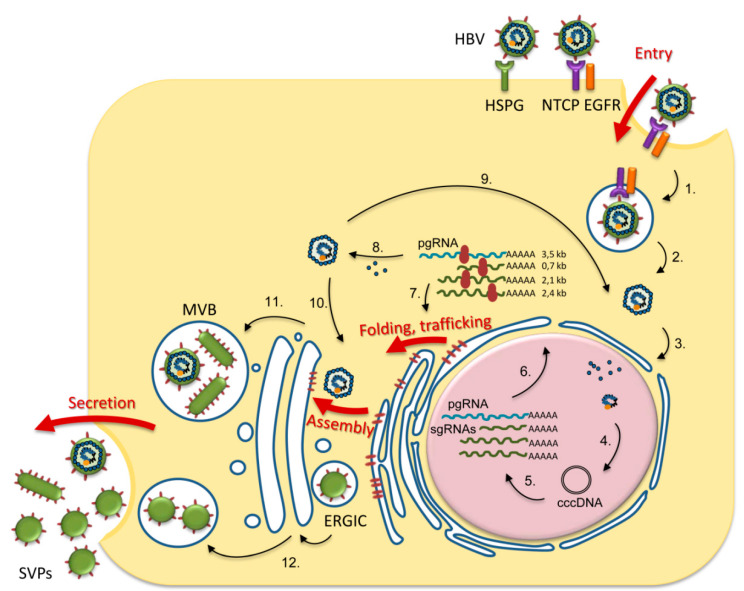
Overview of N-glycosylation roles in the HBV lifecycle. HBV entry begins with virion attachment to heparan sulfate proteoglycan (HPSG) followed by specific interaction with sodium taurocholate cotransporting polypeptide (NTCP)–epidermal growth factor receptor (EGFR) complex on the hepatocyte membrane, leading to endocytosis (1.). The HBV nucleocapsid is released from the endosomes (2.) and further transported to the nucleus (3.), where the viral genome is repaired by cellular enzymes to form covalently closed circular (ccc) DNA (4.). cccDNA is the template for transcription of pregenomic (pg) and subgenomic (sg) RNAs (5.), which are transported to the cytosol (6.). The viral proteins (polymerase, core, HBe, HBx, S, M, L) are synthesized from pg and sgRNAs (7.). The pgRNA and the viral polymerase are encapsidated, followed by pgRNA reverse-transcription to DNA (8.). The viral nucleocapsids are either directed to the nucleus for cccDNA amplification (9.) or enveloped to form viral particles (10.). HBV particles and filamentous subviral particles (SVPs) are secreted via multivesicular bodies (MVBs) (11.), while spherical SVPs assembled in ER–Golgi intermediate compartment (ERGIC) are released via the constitutive secretory pathway (12.) [25,26]. The steps in the HBV life-cycle regulated by N-glycosylation/N-glycan processing are depicted by thick red arrows.

Altogether, the early N-trimming events occurring within the ER are central to HBV glycoproteins folding, assembly and trafficking with significant consequences on production of infectious virions (summarized in Figure 3). Although the involvement of the N-linked glycans in virus transport along the Golgi and beyond is less clear, the abundance of other lectin-like proteins, such as ER–Golgi intermediate compartment (ERGIC) marker ERGIC-53 or vesicular integral-membrane protein VIP36 warrants further investigation into a potential role in HBV particles trafficking [39].

## 4. HBV N-glycosylation and the Interaction with the Host Immune System: Implications for Vaccine Development and Diagnosis

Owing to their display at the surfaces of enveloped viruses, N-linked glycans often play instrumental roles during the interaction of the pathogen with the host immune response. Glycans mediate interactions of antigens with immunocompetent cells but also facilitate viral escape by covering neutralizing epitopes or by structurally hindering antigen processing [40].

One of the early mechanisms activated in an infected organism, to counteract the infection, involves pathogen recognition by the innate immune system. The process is mediated by host pattern recognition receptors (PRR), represented by a heterogeneous family of molecules. Of these, the calcium-dependent (C-type) lectins play important roles in sensing highly conserved carbohydrate structures associated with the envelope proteins. An important member of this family is the dendritic cell-specific intercellular adhesion molecule-3-grabbing non-integrin (DC-SIGN), which recognizes high-mannose oligosaccharides exposed on a variety of viruses, such as HCV, HIV, or Ebola [41]. Usually, this process leads to virus capturing and internalization by dendritic cells (DCs). DC activation triggers the adaptive immune response, enabling presentation of processed viral antigens to T lymphocytes, contributing to pathogen clearance [42,43]. However, some viruses have adapted to hijack this entry pathway and use it for their own benefit as an intermediate station to their primary target cells [44].

Interestingly, native HBV particles and SVPs containing complex-type N-linked glycans failed to interact with either DC-SIGN or the liver-expressed counterpart, L-SIGN [45]. In contrast, production of virions with high-mannose oligosaccharides in the presence of kifunensine resulted in DC-SIGN recognition and binding. More recently, the functional consequence of HBV binding to DC-SIGN was assessed by monitoring the DC maturation and activation when exposed to wild-type and highly mannosylated particles [46]. The study indicated that a stronger, DC-SIGN dependent immune activation occurs when DCs are stimulated with HBV exposing high-mannose N-linked glycans on the enveloped proteins. Similarly, an improved cellular response was observed following immunization with HBsAg attached to mannosylated solid lipid nanoparticles as compared to nanoparticles lacking mannose [47].

An interesting hypothesis emerging from these studies is that N-glycan trimming along the secretory pathway may be an important mechanism contributing to the inefficient innate immune response against HBV. Thus, manipulation of N-linked glycosylation of HBV proteins, to facilitate uptake and processing by antigen-presenting cells, may be relevant to the development of novel vaccines with more potent immunogenic properties. Moreover, not only the composition but also the number of the N-linked glycans appears to influence the interaction of the envelope proteins with antigen-presenting cells. Indeed, increasing the density of the N-glycans attached to the S protein by introducing two additional N-glycosylation consensus sites at T116 and G130 resulted in higher antibody titers in mice vaccinated with the corresponding SVPs. The hyper-glycosylated antigens induced earlier and longer-lasting humoral immune responses as compared to the wild-type version, strongly suggesting a glycosylation-dependent interaction with the innate immune system [48].

Changes of the N-glycosylation pattern of HBsAg were also tested in the case of HBV DNA vaccines that are promising alternatives for individuals who fail to respond to the standard, recombinant S protein-based vaccine. Delivery of HBV antigens by means of DNA vaccination was shown to induce protective antibody levels in these patients, possibly by activating both cell-mediated and humoral immune responses [49]. The strong T-cell response obtained by DNA as compared to protein immunization has suggested the utility of the former as immunotherapy for chronically infected HBV patients. Further development of a DNA vaccine has addressed expression of the M protein and the role of N-glycosylation on the immune response induced in vaccinated mice [50]. Interestingly, de-glycosylation of the S domain of this antigen resulted in significant reduction of cell-mediated immune response, while production of anti-S antibodies was not affected [50]. Thus, production of almost equal ratios of glycosylated and non-glycosylated envelope proteins in infected cells is suggestive of a fascinating mechanism adopted by the virus to control the T cell response and achieve chronic infection.

Removal of the conserved preS2 N-linked glycan of the M protein had deleterious effects on both virus particles and M-SVPs [11]. Moreover, N4 glycosylation was later shown to be a crucial determinant of the M-based DNA vaccine efficiency [51]. Therefore, studying an M protein with altered rather than absent N-linked glycans was an alternative approach to further optimize the antigenic features of this immunogen. Indeed, preventing N-glycan trimming by treatment with ER α-glucosidase I inhibitors resulted in proteasome-mediated degradation of M-SVPs, enhanced presentation of viral peptides by Major Histocompatibility Complex Class I (MHC class I) and activation of CD8+ cytolytic T cells (CTLs) in vitro [52]. While the utility of such a pharmaceutical approach is awaiting confirmation in future studies, it becomes apparent that manipulation of the ER-specific N-glycan processing remains an important tool for development of improved HBV immunogens.

Protection against the humoral immune response generated during a physiological infection is another important function of N-glycans. During the course of HBV infection, patients may clear the HBsAg either spontaneously or following antiviral therapy, progressing to the HBsAg seroconversion stage, characterized by detection of protective HBsAg antibodies titers. This is a very important therapeutic endpoint indicating that no active HBV replication occurs in these patients. It is well documented that up to 5% of chronically infected HBV patients are both HBsAg- and anti-HBsAg-positive, suggesting failure of the humoral immune system to mount a protective response in some individuals [53,54,55]. This is likely due to selection of adaptive mutations within the major hydrophilic region (MHR, amino acids 99–169) of the S domain, occurring naturally or under immune system pressure. Interestingly, several of these mutations generate novel N-glycosylation sites [53]. The most frequent mutations altering the HBV N-glycosylation pattern, isolated from infected patients, are summarized in Table 1.

A general feature of these hyper-glycosylated mutants is their reduced affinity for various HBsAg antibodies, which can be restored by de-glycosylation [53,57]. This suggests that these mutants may escape the immune system surveillance even in vaccinated people. This concept is supported by the horizontal transmission of the N-glycosylation variants in recipients of the standard HBV vaccine, as recently demonstrated [53].

The mutations described above could also influence the viral polymerase activity, due to the overlapping nature of the two ORFs in the HBV genome. However, the evidence gathered so far is conflicting. At least in trans-complementary studies, some mutants appeared to increase viral production, possibly by facilitating nucleocapsid envelopment [60], while others had opposite effects [57] (see Table 1). In the last scenario, selection of these mutants indicates that potential loss on viral fitness is outweighed by the benefits on survival under the immune attack, possibly contributing to establishment of chronic infections.

The interaction of HBV N-glycan mutants with the immune response appears responsible for a series of intriguing features of the disease, such as viral reactivation or occult infection.

HBV reactivation is detected in patients with inactive infection or who have cleared the virus previously, either spontaneously, or under immunosuppressive therapy. This often results in more severe disease and higher mortality rates due to liver failure [62]. It is interesting to note that these individuals display a high genetic variability of the HBsAg, a significant proportion bearing mutations introducing additional N-glycosylation sites within the MHR of S [63]. In addition, most of these patients are HBsAg-negative at the onset of HBV reactivation. However, this is due to a poor recognition of the mutant proteins by the HBsAg antibodies in commercial ELISA kits and not to an impaired synthesis/secretion, as recently demonstrated [58] (see also Table 1). Since misfolded proteins are usually retained within cells by the ER protein quality control [15], the altered antigenicity of hyper-glycosylated HBsAg may be attributed to epitope shielding rather than to mutation-induced conformational changes. With this in mind, it is not surprising that N-glycosylation mutations are increasingly reported in individuals with occult HBV infection (OBI), characterized by sustained viral replication in the absence of detectable HBsAg [59,61,64] (Table 1). This observation has great clinical relevance, highlighting the importance of complementary assays for accurate diagnosis of HBV infection in HBsAg-negative patients.

Additional N-glycosylation does not seem to contribute directly to the severity of pathogenesis observed in reactivated HBV infections, as in most cases the virus replication is not significantly altered [58,59]. Therefore, the ability of hyper-glycosylated HBsAg antigens to evade the immune system surveillance is a sufficient benefit for the virus, justifying selection and enrichment of the corresponding mutations in these patients.

## 5. HBV N-glycosylation and the Carcinogenic Potential

HBV is the major etiologic agent of hepatocellular carcinoma (HCC), the third leading cause of cancer mortality in the world [65]. Several factors were proposed to influence HCC development in chronically infected HBV patients, including the viral load, a range of mutations consistently found within the envelope proteins [66,67] and coexistence of HBsAg/anti-HBsAg antibodies [68,69].

As discussed in the section above, a significant number of patients with coexisting HBsAg/anti-HBsAg antibodies are also characterized by the presence of mutations introducing novel N-glycosylation sites within the MHR of the S protein [53]. Interestingly, these mutations are significantly more frequent in HBsAg/anti-HBsAg antibodies-coexisting patients who develop HCC than in those who do not. Moreover, they are often detected several years before HCC onset, raising the question of a direct involvement of N-linked glycans in disease progression [70].

Recently, twenty additional N-glycosylation sites have been reported in HBsAg/anti-HBsAg antibodies-coexistent patients, of which the previously characterized T131N+M133T mutations (Table 1) were clearly the dominant pattern, being detected in more than 50% of cases. Nevertheless, five mutations comprising insertions (112-113 KNA, 114-115 NTSTT, 115-116 INGTST), triple point substitutions (T116N and T131N+M133T) and single point substitutions coupled with deletions (T113N and 114-116 STT deletion) were completely novel. Functional analysis of the newly discovered mutations was further performed in transfected HepG2 cells. However, neither cell proliferation nor migration was affected in cells expressing HBV-S with additional N-linked glycans [70].

Further studies are needed to understand whether the N-glycosylation mutants described above play a specific role in HCC development. Alternatively, they may be predictive markers associated with an increased carcinogenic risk in chronically infected HBV patients. Both scenarios are clinically relevant and deserve deeper scrutiny. Particular attention should be paid on the ability of these mutants to induce ER stress in hepatocytes in the long term. This is a well-established HCC trigger, as suggested by earlier work on secretion-incompetent, truncated HBV M and L proteins [71].

## 6. Conclusions

The highly conserved and atypical N-glycosylation pattern of the HBV envelope proteins, quite unique among enveloped viruses, has attracted much scientific interest in the last decades. Owing to the great technological development of glycan analysis and of cellular systems supporting full HBV lifecycle, we are now able to understand, at least in part, the multiple facets of N-glycosylation and the role played in the HBV biology. The pre-S2 N-linked glycan is central to M protein folding, assembly and secretion, rendering this protein extremely sensitive to N-glycosylation and N-glycan trimming inhibitors. Due to M oligomerization with L and S, the key determinants of HBV assembly, secretion and infectivity, this vulnerability is transferred to the entire HBV particle, although M itself is not essential in these processes. In contrast, the common N-linked glycan of the S domain plays dual roles during the HBV interaction with the host immune system, enhancing the immunogenicity of the viral antigens on the one hand and occluding neutralizing epitopes at the MHR level on the other hand. Altogether, these functions illustrate the complexity of HBV glycobiology and should be exploited in the future for the designing and development of vaccines and therapies with improved efficacy against HBV.

## Figures and Tables

**Table 1:** Characterization of HBV N-glycosylation mutations isolated from chronically infected patients.

Mutation	Glycosylation	Properties	Reference
T123N	Yes	Poor recognition by HBsAg antibodies (Abs) Triggers anti-HBsAg humoral response Strongly reduces assembly of HBV (genotype C)	[56]
		Significantly reduced reactivity with commercial HBsAg ELISA kits Able to induce protective HBsAg Abs titers Normal viral replication	[57]
		Isolated from patients with HBV reactivation Reduced reactivity with commercial HBsAg ELISA kits HBsAg negativity at reactivation No effect on HBV replication (genotype D) No effect on HBsAg secretion	[58]
T115N T117N 114N-insertion S113N+T131N+M133T *	Yes	Isolated from patients with HBV reactivation Reduced reactivity with commercial HBsAg ELISA kits HBsAg negativity at reactivation No effect on HBV replication (genotype D) No effect on HBsAg secretion	[58]
K160N	Yes	Poor recognition by HBsAg Abs Triggers anti-HBsAg humoral response Enhanced cellular immune response Compensates for the negative effect of A159G mutation on HBV production	[56]
G130N **	NE	Weak recognition by a conformational HBsAg Abs	[55]
Q129N	Yes	Decreased binding to HBsAg Abs Increased HBV production in trans-complementary studies	[53]
		Impaired recognition by HBsAg Abs Poor immunogenicity, still protective HBsAg Abs titers Impairs viral replication but not HBsAg secretion	[57]
		Decreased binding to HBsAg Abs (by 70.3%) Increased HBV secretion in trans-complementary studies	*** [59]
T131N/M133T #	Yes	Impaired recognition by HBsAg Abs Poor immunogenicity, still protective HBsAg Abs titers Normal viral replication	[57]
		Decreased binding to HBsAg Abs (by 84.5%) Increased HBV secretion in trans-complementary studies	*** [59]
T131N #	Yes	Decreased binding to anti-HBsAg Abs Increased HBV production in trans-complementary studies	[53]
“RPCMNCTI” insertion between residues 126−127	Yes	Moderate decrease of HBsAg Abs binding (by 23.2%) Normal HBV secretion in trans-complementary studies	*** [59]
3 aa insertion between residues 114-115	Yes	Decreased binding to anti-HBsAg Abs Increased HBV production in trans-complementary studies	[53]
M133T #	Yes	Increased virion secretion Restores virion secretion of G119E, G145R, I110M, R169P immune-escape mutants but not recognition by the neutralizing Abs.	[60]
T116N (genotype B)	Yes	No significant change of HBsAg antigenicity	*** [61]
TCT123-125NFT (genotype B) TCT123-125NCT (genotypes B and C)	Yes	Low reactivity against HBsAg Abs Impaired HBsAg secretion	*** [61]
TSM131-133NSS (genotype B) TSM131-133NST/NYT (genotypes B and C)	Yes	No significant change of HBsAg antigenicity	*** [61]
NCT146-148SCT/YCT (genotype C) NCT146-148DCT (genotypes B and C)	No Removes the conserved N146 glycosylation site	No significant change of HBsAg antigenicity -Normal HBsAg secretion	*** [61]
GTS130-132NTS (genotypes B and C)	Yes	No significant change of HBsAg antigenicity	*** [61]
GSS112-114NAT (genotype B) TTS115-117NTS (genotype C)	No	No significant change of HBsAg antigenicity	*** [61]

* introduces two N-glycosylation sites, both occupied; ** the isolated clone bears additional mutations within the MHR; *** isolated from a patient with occult hepatitis B infection (OBI); # introduces one N-glycosylation site at 131N; NE, not established.
